# Full-length transcript sequencing accelerates the transcriptome research of *Gymnocypris namensis*, an iconic fish of the Tibetan Plateau

**DOI:** 10.1038/s41598-020-66582-w

**Published:** 2020-06-15

**Authors:** Hui Luo, Haiping Liu, Jie Zhang, Bingjie Hu, Chaowei Zhou, Mengbin Xiang, Yuejing Yang, Mingrui Zhou, Tingsen Jing, Zhe Li, Xinghua Zhou, Guangjun Lv, Wenping He, Benhe Zeng, Shijun Xiao, Qinglu Li, Hua Ye

**Affiliations:** 1 Key Laboratory of Freshwater Fish Reproduction and Development (Ministry of Education), Southwest University College of Animal Sciences, Chongqing, 402460 China; 2 Key Laboratory of Aquatic Science of Chongqing, 400175 Chongqing, China; 3 Department of Computer Science, Wuhan University of Technology, Wuhan, 430070 China; 4 Institute of Fisheries Science, Tibet Academy of Agricultural and Animal Husbandry Sciences, Lhasa, 850000 China

**Keywords:** Gene expression, RNA sequencing

## Abstract

*Gymnocypris namensis*, the only commercial fish in Namtso Lake of Tibet in China, is rated as nearly threatened species in the *Red List of China’s Vertebrates*. As one of the highest-altitude schizothorax fish in China, *G. namensis* has strong adaptability to the plateau harsh environment. Although being an indigenous economic fish with high value in research, the biological characterization, genetic diversity, and plateau adaptability of *G. namensis* are still unclear. Here, we used Pacific Biosciences single molecular real time long read sequencing technology to generate full-length transcripts of *G. namensis*. Sequences clustering analysis and error correction with Illumina-produced short reads to obtain 319,044 polished isoforms. After removing redundant reads, 125,396 non-redundant isoforms were obtained. Among all transcripts, 103,286 were annotated to public databases. Natural selection has acted on 42 genes for *G. namensis*, which were enriched on the functions of mismatch repair and Glutathione metabolism. Total 89,736 open reading frames, 95,947 microsatellites, and 21,360 long non-coding RNAs were identified across all transcripts. This is the first study of transcriptome in *G. namensis* by using PacBio Iso-seq. The acquisition of full-length transcript isoforms might accelerate the transcriptome research of *G. namensis* and provide basis for further research.

## Introduction

The Tibetan Plateau, a harsh environment with an average altitude of 4,500 m, is home to the highest and largest high-altitude lakes in the world^1,2^. The area of lakes on the Tibetan Plateau are more than 50,900 km^2^, and 1,091 lakes are larger than 1.0 km^2,3^. With an area of 1,920 km^2^ and an altitude of 4,718 m, Namtso Lake in the North Tibet is the highest great lake in the world and is the second biggest salt water lake in China^4,5^. As a highly natural, rare, fragile and representative lake, Namtso Lake imposes many inhospitable living conditions on most of organisms, such as the high pH and alkalinity, severe cold (with an annual average temperature of 0°C, five-month ice-covered period), the low primary productivity and oligotrophic conditions^3–7^. Because of the lower temperatures and oligotrophic conditions of Namtso Lake, only two endemic fish species (*Gymnocypris namensis* and *Triplophysa stewarti*) have been found in the lake^6^. *G. namensis*, the only economic fish in Namtso Lake, is known as one of the highest-altitude schizothorax fish in China and it has strong ability to adapt to the plateau harsh environment^6,8^. However, systematic biological studies on *G. namensis* are chronically lacked due to extremely harsh environments on the plateau^8,9^.

In previous transcriptome studies in other fish of geneus Gymnocypris, such as *Gymnocypris selincuoensis*^10^*, Gymnocypris przewalskii*^11^*, Gymnocypris eckloni*^12^, some progress has been made. In transcriptome studies of *G. selincuoensis*, a full-length reference transcriptome has been generated by using PacBio Iso-Seq and Illumina RNA-seq technologies. But the most of other transcriptome studies used next-generation transcriptome sequencing technologies with short reads. Although next-generation transcriptome sequencing data characterized by short reads have been widely used in modern biological research, recent studies have shown that short-read transcriptome technology faces enormous challenges. For example, it is still insufficiently accurate to reconstruct and quantify complete transcript isoforms after identifying all transcript elements^13,14^. Especially due to its short read length, it is not suitable for the study of specific biological problems such as the determination of complex genome regions, the detection of homologous isomers and methylation^15^. The complexity of transcriptome plays an important role in determining gene coding potential and regulating gene expression through multiple mechanisms^16–18^. The next generation transcriptome technology of short reads is hard to address this kind of issues, therefore many studies have been devoted to the sequencing technology of long reads^14,19–21^. Single molecule real-time sequencing (SMRT) technology developed by PacBio company is a long-read sequencing technology that overcome many defects of next-generation sequencing technology^15^. These long reads data can cover different exon connections to obtain full-length transcripts^14,15^. The combination of the two technologies can effectively overcome their respective shortcomings in order to obtain longer and more accurate transcript information for biological research^15,22,23^. At present, this method has been used in many animals and plants, such as *G. selincuoensis*^10^, Jiejie wheat^22^, corn^24^ and American beaver^25^, however, there are still few studies in aquatic animals.

In this study, the two technologies of PacBio and Illumina sequencing were combined to analyze the transcriptome of *G. namensis* with the pooled tissues. The major objective of this study was to generate and annotate a full-length reference transcriptome. Based on the obtained transcripts information, we performed transcript functional annotation, microsatellites analysis, coding sequence prediction, and lncRNA prediction, providing valuable and comprehensive gene sequence resource to the research community for the further gene function and environmental adaptation studies.

## Materials and Methods

### Ethics statement

All of the experimental procedures were approved by the ethics committee of Southwest University. The methods involving animals in this study were conducted in accordance with the Laboratory Animal Management Principles of China.

### Sample collection

**Figure 1:**
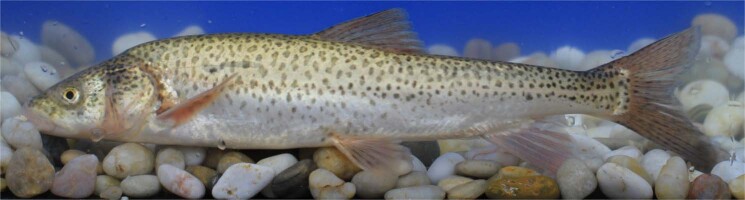
A picture of *Gymnocypris namensis* in Namsto Lake in Tibet.

A female adult *G. namensis* fish (Fig. 1) was collected from Namsto Lake (30°39′52.16″N, 90°17′25.30″E, 4,736 m) in Tibet, and numbered as 0906 (703.1 g body weight). To obtain as many expressed genes as possible, ten tissues (gill, brain, heart, liver, kidney, spleen, intestine, skin, muscle, and blood) were dissected and immediately immersed in liquid nitrogen, then kept at −80 °C.

### RNA extraction

Total RNA was prepared according to the methods of Ye *et al*.^26,27^. Total RNA was isolated from different tissues using a RNAiso Plus Reagent Kit (Takara Biotechnology, Dalian, China) following the manufacturer’s instructions. The purified RNA was dissolved in RNase-free water, with genomic DNA contamination removed using TURBO DNase I (Promega, Beijing, China). The integrity and purity of the total RNA were checked with a Nanodrop 2000C spectrophotometer (Thermo Scientific, Waltham, Massachusetts) and Agilent 2100 Bioanalyzer (Agilent Technologies, Palo Alto, California). Only the total RNA samples with RIN value ≥ 8 were used for constructing the cDNA library in PacBio or HiSeq sequencing^28^.

### PacBio Iso-Seq library preparation and sequencing

In order to construct libraries for PacBio sequencing, 0.2 ug RNA from each of ten tissues of 0906, including gill, brain, heart, liver, kidney, spleen, intestine, skin, muscle, and blood were pooled, resulting in one pooled library. The sequencing library was prepared in terms of the Pacific Biosciences’s Iso-Seq sequencing protocol as described briefly as following: Firstly, a total of 2 μg purified polyA(+) RNA was reversely transcribed into cDNA with the method of the SMARTer PCR cDNA Synthesis Kit (Takara Biotechnology, Dalian, China) using Oligo-dT primers. After a round amplification with polymerase chain reaction (PCR), the products were size selected using the BluePippin™ Size Selection System (Sage Science, Beverly, MA). Secondly, each SMRT bell library was constructed utilizing size-selected cDNA with the Pacific Biosciences DNA Template Prep Kit 2.0. The combining of SMRT bell templates to polymerases was conducted using the DNA/Polymerase Binding Kit. Sequencing was implemented on the PacBio sequel platform by Frasergen Bioinformatics Co., Ltd. (Wuhan, China).

### Illumina transcriptome libraries preparation and sequencing

The mRNA was purified from the total RNA using poly-T oligo-attached magnetic beads. The short-reads RNA sequencing libraries used to correct the FLNC (Full-length non-chimeric) reads were constructed with the TruSeq RNA Sample Prep Kit (Illumina, San Diego, CA) with multiplexing primers, on the basis of the manufacturer’s protocol, which included a 300-bp size selection step. The cDNA was purified using a QiaQuick PCR extraction kit (Qiagen, Inc., Hilden, Germany). The suitable fragments after end repair, adapter ligation, and agarose gel electrophoresis filtration were selected as templates for PCR amplification. Libraries were sequenced on 1 lane of Illumina HiSeqX ten (Illumina Inc., San Diego, CA, USA). The raw sequencing reads generated by the Illumina Hiseq X ten platform were processed using in-house perl scripts, following the quality control standards of previous studies^29^. Clean reads used for SMRT error correction were obtained after removing reads containing poly-N, adapters, and low-quality reads. Raw reads are available in the NCBI SRA under the Bioproject accession number PRJNA562739.

### PacBio data analysis

The raw sequencing data generated by the PacBio Sequel platform were processed with the standard Iso-Seq protocol (https://github.com/PacificBiosciences/IsoSeq_SA3nUP). In short, we used the software SMRT Link to perform data preprocessing and filtering with the following main parameters: minimum subread length = 300, maximum subread length = 15,000, minimum number of passes = 3, minimum predicted accuracy = 0.8, minimal read score = 0.65, minimum accuracy of polished isoforms = 0.99. Firstly, circular consensus sequence (CCSs), or reads of insert sequence (ROIs) were generated from subread BAM files which were converted by raw sequencing reads. By detecting the presence of chimera sequence, sequencing primer and 3′ terminal poly-A sequence, the CCS sequences were classified into full length, non-chimeric ROIs and non-full length, non-chimeric ROIs. FLNC sequences were determined depended on the existence of the 5′-adaptor sequence, the 3′ adapter sequence and poly (A) tail. Next, we used the ICE (Iterative Clustering and Error Correction) tool of cluster module in the SMRT Link software to cluster and polish multiple FLNC sequences from the same isoform to obtain the non-redundant isoforms sequence. After the isoform sequences were further polished by the non-full-length non-chimeric sequence with the Quiver tool in the SMRT Link software, we obtained the polished isoforms sequence. The polished isoforms were subjected to secondary sequence clustering by cd-hit-est software and further remove the sequence redundancy. The sequence obtained in this step was the final non-redundant isoform sets which were used for all subsequent analysis. Due to the frequency of mismatches and nucleotide indels are much higher in PacBio Iso-Seq reads than in shorter high-throughput sequencing, the polished FLNC were corrected by Illumina RNA-Seq reads, using proovread, a hybrid correction pipeline with default parameters^30^. The final full-length transcript sequences were generated after the error-corrected FLNC sequences were clustered and reduced redundancy using cd-hit-est software with local sequence identity threshold of 99%. The alignment ration (sequence length divided by the alignment length) for sequences should be larger than 90%.

### Functional annotation of transcripts

The non-redundant transcript isoforms were annotated by running diamond (v0.8.33.95) and Blast2GO^31^ searches against five public databases, including NCBI non-redundant protein database (Nr), the euKaryotic Ortholog Groups (KOG), the Kyoto Encyclopedia of Genes and Genomes (KEGG), Gene Ontology (GO), and Swiss-Prot. The cut-off values of the searches for Nr, KEGG, GO, Swiss-Prot was 1e^−5^, and 1e^−2^ for KOG.

### Evolutionary analysis using *G. namensis* transcripts

The protein-coding genes were extracted from the genome and annotation in Ensembl^32^ and NCBI database. Only longest transcripts were used to represent the gene. Those gene sequences were blast by an all-vs-all manner using Blast utilities, and the gene families were clustered using the OrthoMCL pipeline^33^ with default settings. Genes clustered into the same gene families were orthologous genes and the single-copy orthologs across all the species were selected for the phylogenetic analysis. The gene sequences were then aligned by MAFFT^34^ v7.407. Phylogenies of genes in one family were inferred by Maximum Likelihood in RAxML^35^ with GTRGAMMA model. The phylogenies and alignments were used to detect positive selection using CodeML program of the PAML^36^ software (v4.9) package with the branch-site model and a FDR threshold of 0.01. The functional enrichment with respect to GO function or biological pathways of genes under positive selection was performed using hypergeometry distribution in R package.

### LncRNA prediction

Based on previous annotation results, the transcripts with no annotation information in the protein database were assessed its coding potential by using coding potential assessment tool (CPAT)^37^. After filtering the sequences with a coding potential greater than a certain cutoff (based on the intersection of the sensitivity curve and the specificity curve of coding probability Cutoff) or length <200 bp, the rest of the transcripts were selected as lncRNA candidates.

### SSR detection

MISA software (http://pgrc.ipk-gatersleben.de/misa/) with default parameters was used to predict the SSR markers in the transcriptome of *G. namensis*.

### Prediction of coding sequences (CDS)

TransDecoder v3.0.1 software^38^ was used to predict open reading frames (ORFs) of the non-redundant transcript isoforms with a minimum CDS of 100 bp. To enhance the sensitivity of the predicted ORFs, the predicted protein sequences of the possible ORF translations were identified by BlastP with e-values of 1e^−5^ alignment to the Swiss-Port protein database for homologous protein identification, and the protein domains were identified by searching the Pfam database with Hmmscan software^39^. Based on homologous proteins and protein domains, ORFs with homology to known protein libraries or identified to the same protein domain were preserved.

## Results

### The output of PacBio sequencing and error correction

A multiple-tissue hybrid library was sequenced on the PacBio Sequel platform using the C3 reagents with 2 SMRT cells, 1,012,473 polymerase reads were generated (Table 1). After preprocessing, 615,874 circular consensus sequence (CCS) reads were obtained (Table 1). The average length of CCS in 2 SMRT cells is 1,663 bp, and 1,690 bp, respectively (Table 1). Further analysis, we obtained 270,520 (including 267,490 FLNC with an average length of 1,542 bp) and 296,536 (including 292,946 FLNC with an average length of 1,568 bp) full-length reads from 2 SMRT cells (Table 1). The FLNC sequences were clustered and remove redundancy, using the ICE tool of the PacBio SMRT link software to obtain a non-redundant isoform sequence set. Further, the Quiver tool of the SMRT link software was used to correct the above non-redundant isoform sequence set by means of the non-full length non-chimeric short sequence, 319,044 high-quality isoforms were obtained (Table 2).

**Table 1:** PacBio Iso-seq output statistics.

Libraries	09061	09062
Polymerase reads	492,350	520,123
Mean length of polymerase reads	17,327	17,542
Polymerase reads N50	34,250	33,250
Subreads	5,790,289	6,032,472
Mean length of subreads	1,396	1,434
Number of circular consensus sequence reads (CCS)	295,248	320,626
Mean length of CCSs	1,663	1,690
Total bases of CCSs	491,225,617	542,141,219
Number of reads with 5′ adapter sequence	282,632	308,526
Number of reads with 3′ adapter sequence	284,198	309,794
Number of poly-A reads	281,763	307,616
Number of full-length reads	270,520	296,536
Number of full-length non-chimeric reads	267,490	292,946
Mean full-length non-chimeric read length	1,542	1,568
Full-length percentage (FL%)	54.94	57.01

**Table 2:** Summary statistics of the isoforms.

Isoform types	Polished high-quality isoforms	Short reads corrected isoforms	Non-redundant isoforms
Total bases	488,919,715	488,137,232	228,095,655
Total number	319,044	319,044	125,396
Average length	1,532	1,530	1,819
Maximum length	11,351	11,289	11,289
Minimum length	132	132	132
Median length	1,315	1,312	1,577
N50	1,553	1,552	2,044

**Figure 2:**
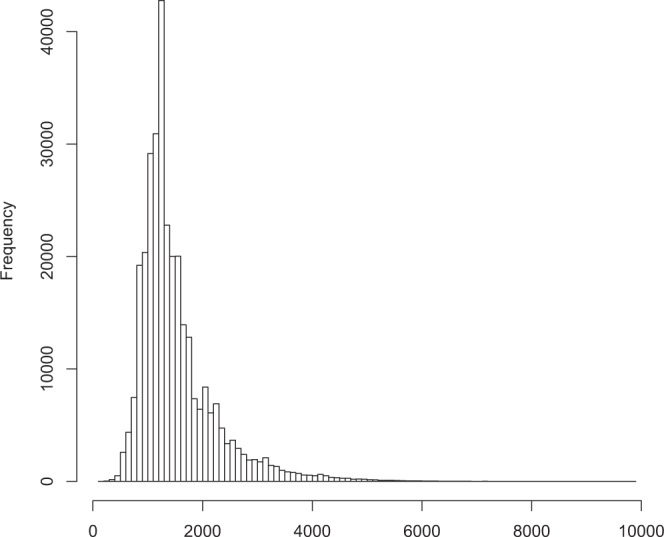
Length distribution of transcript isoforms. The x-axis represents the transcript isoforms length, the y-axis represents the number of the transcript isoforms.

We used a total of 207.30 million short-reads obtained from Illumina sequencing to correct the 319,044 FLNC reads. After correcting the above high-quality isoforms by means of the proovread error-correcting software^30^, the error correction FLNC sequences were further clustered and removed redundant by using cd-hit-est software, and finally 125,396 non-redundant isoforms were obtained. The lengths of non-redundant isoforms ranged from 132 to 11,289 bp with an average length of 1,819 bp, and its median length and N50 was 1,577 and 2,044 bp, respectively (Table 2). The majority of transcript isoforms (264,548, 82.92%) exceeded 1,000 bp (Fig. 2). The non-redundant transcript isoforms were used in following analyses.

To probe the possible contamination of transcripts generated in this study, we searched the transcripts against NCBI NT database and found that the top three organism sources for the best hits were *Sinocyclocheilus* (83%), *Cyprinus* (16%) and *Danio*(0.5%) genus, suggesting no significant contamination was detected among the transcripts.

### Functional annotation of transcript

**Figure 3:**
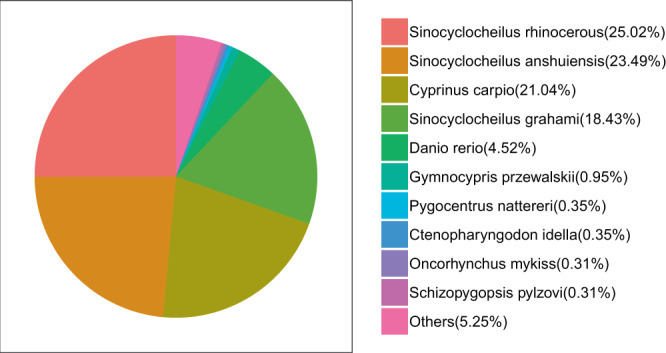
The species identified by homology search against the NCBI NR databases. Note that only the best hits for transcripts are covered in the analysis.

In total, 103,286 transcripts were annotated at least one public database, including 49,138 (39.19%) in KOG, 69,446 (55.38%) in KEGG, 103,213 (82.31%) in NR, 63,926 (50.98%) in GO, and 90,820 (72.43%) in Swiss-Prot. 22,110 (17.63%) transcripts weren’t annotated in above public databases. Based on 103,213 high quality transcripts which were annotated in NR database, homology search was conducted to identify the species with highly similar sequences deposited in database. Around 25.02% of transcript sequences were aligned to *Sinocyclocheilus rhinocerous*, followed by *Sinocyclocheilus anshuiensis* (23.49%), and *Cyprinus carpio* (21.04%) (Fig. 3), which was consistent with the close evolutionary relationship among those species.

**Figure 4:**
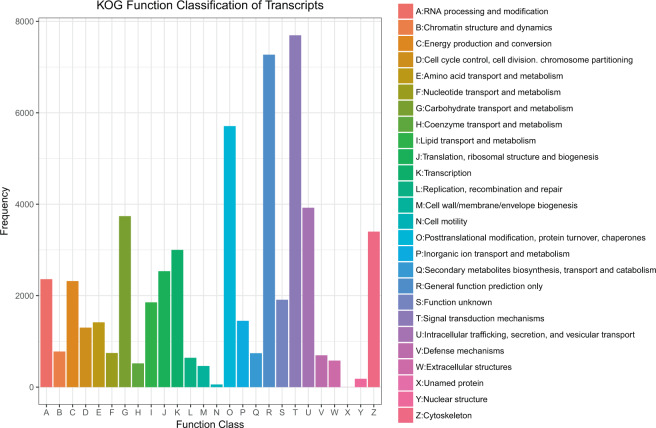
KOG function classification of transcripts of *G. namensis*. Letters on the x-axis represents different KOG categories, as shown detail on right legend, the y-axis represents the number of the transcripts.

The potential functions of all full-length transcripts were predicted using the KOG database, 49,138 transcripts were grouped into 26 KOG classifications (Fig. 4). The largest number of category was the Signal transduction mechanisms (7,696, 15.66% of the matched transcripts), followed by the General function prediction only (7,271, 14.80%), the post-translational modification, protein turnover, chaperones (5,710, 11.62%), intracellular trafficking, secretion, and vesicular transport (3,924, 7.99%), carbohydrate transport and metabolism (3,739, 7.61%) (Table S1, Fig. 4).

**Figure 5:**
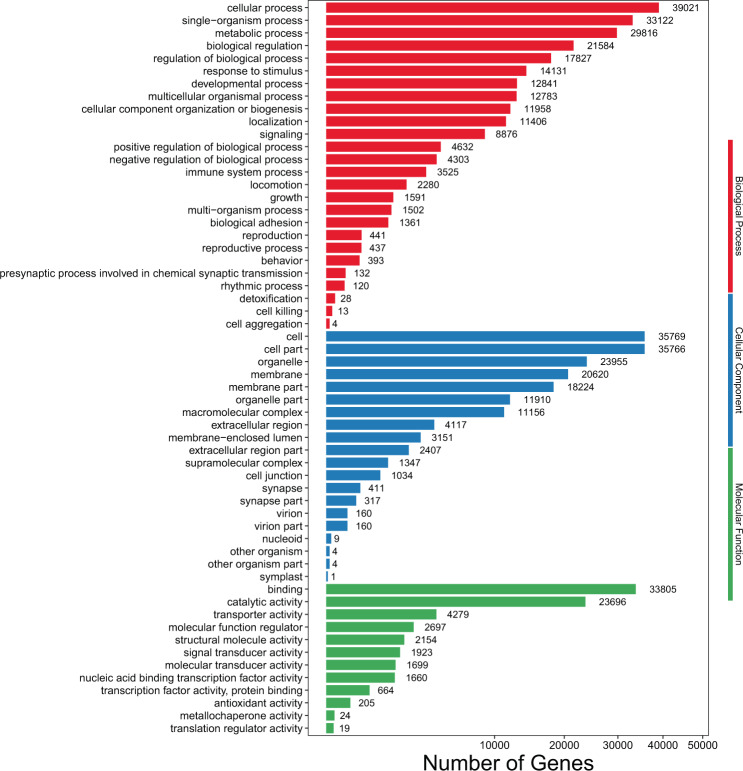
GO annotation of *G. namensis* transcriptome. The x-axis represents the number of genes, the y-axis represents different GO categories.

After GO annotation was performed on all full-length transcripts, the successfully annotated transcripts were classified according to the next level of GO Biological Process (BP), Cellular Component (CC), Molecular Function (MF). The classification results are shown in Fig. 5. GO analysis revealed 63,926 transcripts were assigned to 58 level-2 GO terms, of which 48,924 transcripts (76.53%) were assigned to biological process, 48,926 transcripts (76.54%) were assigned to molecular function, and 46,150 transcripts (72.19%) were assigned to cellular component (Table S2; Fig. 5). The largest subcategory in biological process was “cellular process” (39,021 transcripts), which was represented by 61.04% of the GO annotated transcripts. In the cellular component category, the top 2 subcategories were “cell” (35,769 transcripts) and “cell part” (35,766 transcripts), making up 55.95% and 55.95% of the GO annotated transcripts. “Binding” (33,805 transcripts; 52.88% of the GO annotated transcripts) was the most abundant subcategory in the molecular function (Fig. 5).

**Figure 6:**
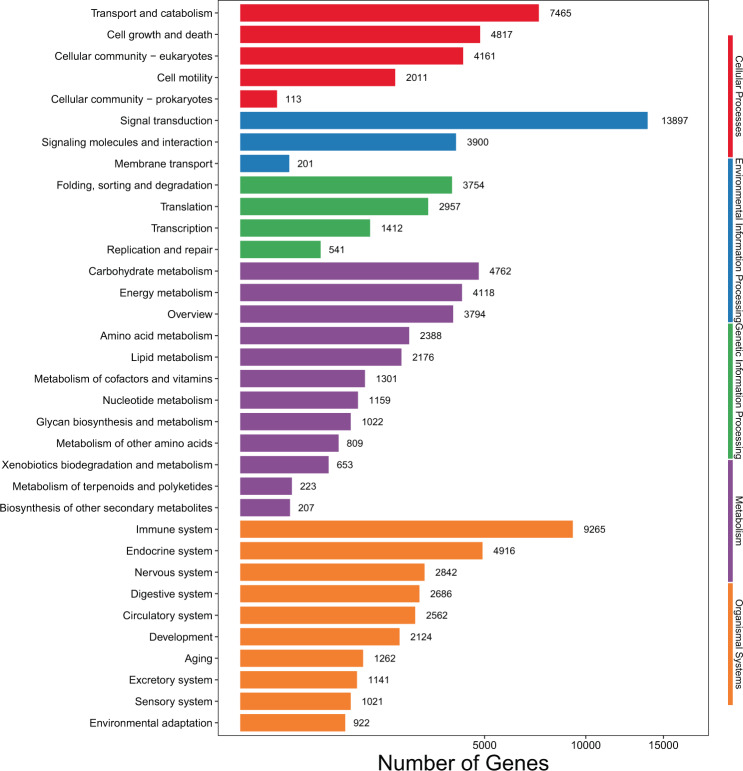
Identified KEGG pathways of transcript isoforms. The x-axis represents the number of genes, the y-axis represents different KEGG pathways.

To obtained the overall biological function of *G. namensis* transcriptome, the full-length transcripts were further annotated by mapping these sequences into reference canonical pathways in KEGG using KOBAS 3.0^40^. A total of 69,446 (55.38%) transcript isoforms were mapped to KEGG Orthology (KO) categories and grouped into 298 signaling pathways, and the number of transcript isoforms in different signaling pathways ranged from 1 to 3,235 (Table S3). The annotated pathways were grouped into five level-1 KO terms, according to the number of annotated transcripts, which in turn were organismal systems, metabolism, cellular processes, environmental information processing, and genetic information processing (Fig. 6). Signal transduction (13,897, 20.01%), Immune system (9,265, 13.34%) and Transport and catabolism (7,465, 10.75%) were top three of the most level-2 KO terms (Fig. 6).

### The coverage of current transcriptome

To provide useful information for the coverage of the recent transcriptome, we performed two analyses using the isoforms data generated from this work. We evaluated the proportion of non-redundant isoforms found in 09061 and 09062 libraries by cd-hit-est, since the statistics for the proportion of non-redundant isoforms (125,396 isoforms) found in 09061 and 09062 libraries could reflect the coverage of current transcriptome. We identified 101,983 and 103,006 non-redundant isoforms from the 09061 and 09062 libraries, representing about 81.3% and 82.1% of all non-redundant isoforms, respectively. Of them, 90,479 isoforms were identified in both libraries, accounting for 72.2% of all non-redundant isoforms. Therefore, the majority of the transcripts were identified in this work, however, there are still isoforms not covered in the current libraries.

Meanwhile, we performed homology search against transcripts recently reported for *G. namensis*, *G. selincuoensis*, *G. pzewalskii* and *G. eckloni*^41^. After searching the gene sequences to the public transcriptome data, we found that more than 96% of isoforms generated from PacBio sequencing could hit homologs in NGS-based transcriptome of *G. namensis*, *G. selincuoensis*, *G. pzewalskii* and *G. eckloni*. However, we found that only 68–74% transcripts from NGS-based transcriptome could hit homologs in the PacBio-based transcriptome (Table S4). Two-fold reasons could be attributed to the relatively low hit ratio for NGS-based transcriptome to PacBio-based data. Firstly, isoforms form PacBio-based transcriptome were filtered through strict criterion of the existence of the 5′-adaptor sequence, the 3′ adapter sequence and poly (A) tail. Therefore, the absolute majority of the isoforms from the Pacbio-based transcriptome were protein-coding genes. However, we found that transcripts in the NGS-based transcriptome that failed to hit PacBio-based data were likely to be non-coding (Table S5). Secondly, the NGS-based transcriptome was more fragmented than PacBio-based one. The mean and N50 length of our transcriptome built using PacBio platform were much higher than the latest *G. namensis* transcriptome from NGS sequencing. Therefore, shorter gene sequences might also reduce the hit ratio during the homolog searching (Table S6).

### Evolution analysis using *G. namensis* transcripts

The transcripts generated in this work were further used to investigate the evolutionary relationship of *G. namensis* and related fish species. The orthologous genes were identified from gene family clustering for *G. namensis* and *Takifugu rubripes*, *Danio rerio*, *Ctenopharyngodon idellus*, *C. carpio* and *S. rhinocerous*. The single-copy orthologous genes were used for the evolution analysis. As a result, *G. namensis* exhited closer relationships with *Sinocyclocheilus* genus and *Cyprinus* genus **(**Fig. S1**)**. Based on comparative analysis for orthologous genes pairs among *G. namensis*, *C. carpio* and *S. rhinocerous*, natural selection detection was detected from 42 genes in *G. namensis* (Table S7). We also performed the phylogenetic analysis using *Gymnocypris* transcriptome data from the latest data^41^. Based on 346,029 sites from 597 single-copy orthologous genes, we generated the phylogenetic relationship of *Gymnocypris* species with other teleost. Consistent with previous study, we found that all *Gymnocypris* species were group into one clade. In addition, we detected 31 positively selected genes for *G. namensis* after adding *G. selincuoensis*, *G. pzewalskii* and *G. eckloni*, among which 22 were overlapped with the analysis without other *Gymnocypris* species (Table S7). Those results showed that natural selection has already exerted on the common ancestor of *Gymnocypris* genus.

### LncRNA prediction

Finally, 21,360 lncRNA were obtained in the 125,396 transcripts. The length of lncRNA ranged from 201 to 11,289 bp, with the majority (å 87.12%) having a length ≤2000 bp. The mean length was 1,819 bp, and there were 17,664 lncRNA which shorter than the mean length.

### SSR prediction

A total of 125,396 sequences with a total length of 228,095,655 bp were subjected to SSR prediction. As a result, 18,084 sequences contain more than one SSR marker. The number of SSRs present in compound formation was 29,972, and the remaining 55,137 are simple SSRs (Table 3) and 10,838 are interrupted SSRs. Most of simple SSRs identified were mono-nucleotide repeats (32,274; 58.53%), followed by the di-nucleotide repeats (16,261; 29.49%), tri-nucleotide repeats (6,014; 10.91%), tetra-nucleotide repeats (498; 0.90%), hexa-nucleotide repeats (68; 0.12%), and penta-nucleotide repeats (22; 0.04%) (Table 3).

**Table 3:** Repeat numbers and unit length distribution of putative pure SSR markers in the transcriptome.

Repeat number	Motif length						Total	Percent (%)
	mono	Di	Tri	Tetra	Penta	Hexa		
5	0	0	3,455	227	37	18	3,737	6.78
6	0	5,419	1,326	104	8	2	6,859	12.44
7	0	2,815	616	23	6	1	3,461	6.28
8	0	1,878	298	16	1	0	2,193	3.98
9	0	1,301	135	9	1	0	1,446	2.62
10	9,782	1,030	71	14	0	0	10,897	19.76
11	5,170	700	37	6	2	0	5,915	10.73
12	3,252	507	17	10	0	0	3,786	6.87
13	2,253	457	17	3	5	0	2,735	4.96
14	1,534	316	3	20	1	0	1,874	3.40
15	1,035	210	12	5	1	1	1,264	2.29
16	719	195	6	2	1	0	923	1.67
17	538	193	3	4	0	0	738	1.34
18	435	135	11	6	0	0	587	1.06
19	371	87	0	2	2	0	462	0.84
≥20	7,185	1,018	7	47	3	0	8,260	14.98
Total	32,274	16,261	6,014	498	68	22	55,137	100.00
Percent (%)	58.53	29.49	10.91	0.90	0.12	0.04	100.00	

### Prediction of coding sequences

**Figure 7:**
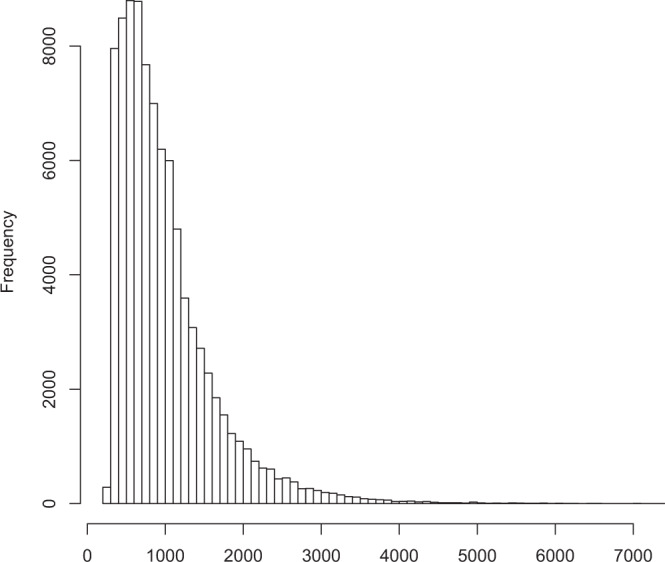
Length distribution of the coding sequence of complete ORFs. The x-axis represents the coding sequence length, the y-axis represents the number of predicted ORFs.

Using TransDecoder v3.0.1 software, 89,736 ORFs were identified. The distribution of the coding sequence lengths of ORFs is shown in Fig. 7.

## Discussion

Namtso naked carp (*G. namensis*) is a unique economic fish in Namtso Lake, but its biological characterization is still unclear. Recently, it has undergone a drastic fishery resources recession due to environmental pollution and commercial exploitation^42^. Therefore, it is extremely urgent to protect germplasm resource of *G. namensis*. With the aim to obtain a general and broader transcriptome resource of *G. namensis*, we sequenced the multi-tissue pooled RNA libraries. In this study, we employed PacBio SMRT sequencing to generate 17.65 Gb clean data, including 615,874 CCS and 319,044 polished isoforms. After correcting the above isoforms with short-reads from Illumina sequencing and removing redundant sequences, we obtained 125,396 high-quality non-redundant full-length transcripts for *G. namensis*. 95,947 SSRs and 89,736 protein-coding sequences were identified. A total of 21,360 lncRNAs were predicted by CPAT. Functional annotation of transcripts indicated that 103,286 transcripts were annotated into at least one functional database. These full-length transcripts obtained in this study would be facilitated further research on *G. namensis* and other schizothorax.

The homology search uncovered that 25.02% sequences exhibited homology hits in NR search to the sequences of *S. rhinocerous*, 23.49% to the sequences of *S. anshuiensis*, 21.04% to the sequences of *C. carpio*. *S. rhinocerous* and *S. anshuiensis* belong to the subfamily of barbinae^43,44^. Most of ichthyologists have shown that the subfamily of the schizothoracinae originates from the subfamily of barbinae^44,45^. The evolution analysis using orthologous genes among those species also revealed that *G. namensis* was close to *Sinocyclocheilus* and *Cyprinus* genus. This result confirmed that evolutionary relationship is very close between *G. namensis, S. rhinocerous*, *S. anshuiensis*, and *C. carpio*, which was consistent with previous researches, and meet the fact that four species belong to the Cyprinidae family^43,46,47^.

Based on the comparative analysis, natural selection has acted on 42 genes for *G. namensis*, comparing to low altitude fish species of *C. carpio* and *S. rhinocerous*. The functional analysis of those selected genes were enriched on the biological pathway of Mismatch repair and Glutathione metabolism, which might reflected the adaptation requirement of high UV radiation for *G. namensis* in the high altitude habitat. The enrichment on the Mismatch repair was consistent with previous transcriptome analysis for *G. selincuoensis*, the other high altitude fish species^10^. MutL Homolog 3 (*mlh3*) (transcript ID of i3_LQ_sample47d422 | c6501/f1p0/3603.p1) and mitochondrial genome maintenance exonuclease 1(*mgme1*) (transcript ID of i1_LQ_sample47d422 | c51979/f2p0/1450.p1), endonuclease IV (*denB*) (transcript ID of i2_LQ_sample47d422 | c26034/f1p0/2295.p1) genes were found to be selected for *G. namensis*. Those genes play an important role in the repair processes after DNA damage and *mlh3* has been reported in the endometrial carcinoma and colorectal cancer. Although we identified different genes in this work, the functions of identified selected genes for DNA repairing were similar. In addition, we identified that glutathione synthase (*gss*) and glutathione S-transferase (*gst*) (transcript ID of i1_LQ_sample47d422 | c2296458/f1p11/1401.p1) genes that were selected for *G. namensis*. Those two genes are key genes in the Glutathione metabolism, implying those genes might contribute to the overoxidation stress response for *G. namensis* under the UV exposure.

Compared with the Illumina short-read sequencing, PacBio SMRT sequencing is more suitable for transcriptome study of non-model animals without reference genome, which could obtain full-length transcripts without assembly^48–50^. PacBio SMRT sequencing has been successfully applied in some researches in aquatic animals^28,51–54^, and provides more comprehensive information of transcriptome, including lncRNAs, alternative splicing, and novel genes. However, this technology has not been applied in *G. namensis*. Recently, Feng *et al*., used PacBio Iso-Seq and RNA-seq to obtain the high quality full-length transcriptome of *G. selincuoensis*, the average length and N50 length of full-length transcripts reached 3,509 and 3,870 bp, much longer than that of the de novo assembled transcripts of Illumina RNA-seq for the same fish species (815 and 1,479 bp). Other indicators, such as the percentage of annotated transcripts, are also much higher than that observed in other fishes with RNA-seq^10^. Similarly, compared with the results of transcriptome in the schizothoracinae using the Illumina short-read sequencing, we obtained a longer average transcript length and N50 length (1,819 and 2,044 bp). The average lengths of transcripts or unigenes obtained in the previous studies in some schizothoracinae were 513–1,323 bp^1,29,55–57^. It is also notable that the mean and N50 length of our transcriptome (1,819 and 2,044 bp) built using PacBio platform was much higher than the latest *G. namensis* transcriptome (1,267 and 1,825 bp) from NGS sequencing^41^. Longer isoforms implied higher sequence integrity for the species, which was rather important for the following gene function and evolutionary studies. Compared with the results of full-length transcriptome of *G. selincuoensis*, the average transcripts length and N50 length (1,819 and 2,044 bp) in this study was shorter than that of in transcriptome of *G. selincuoensis*. There are many reasons for this difference. One possible reason is that the two studies used different sequencing platforms. Another possible reason is that different parameters were used in the data analysis process.

As a novel kind of non-protein coding RNA longer than 200 nucleotides, lncRNAs play important roles in many biological and pathological processes, such as immune responses, cell cycle control, splicing, differentiation, and epigenetic regulation^58,59^. However, no lncRNA in *G. namensis* have previously been reported. Here, we firstly identified 21,360 lncRNA in the *G. namensis* transcriptome, which will be useful for further research of *G. namensis*, such as plateau adaptability, immunology, and epigenetics.

In conclusion, we used PacBio Iso-seq and Illumina short read sequencing to obtain a comprehensive full-length transcriptome of *G. namensis*. To our best of knowledge, this is the first study of whole transcriptome in *G. namensis* by using PacBio Iso-seq. The acquisition of full-length transcript isoforms makes it is more accurate and reliable to gene annotation, development of molecular marker, and lncRNA prediction. Therefore, our comprehensive full-length transcriptome of *G. namensis* provide an important resource for future research of functional gene, molecular markers, molecular events, and signaling pathways. Finally, this study will provide support for the genomic research on mechanism of plateau adaptability in this species in the future.

## Supplementary information

Supplementary information.

Table S3.

Table S7.
