# Supplementary material for: Full-length transcript sequencing accelerates the transcriptome research of Gymnocypris namensis, an iconic fish of the Tibetan Plateau
Source: Sci Rep. 2020 Jun 15;10:9668. doi: 10.1038/s41598-020-66582-w (PMC7296019; doi:10.1038/s41598-020-66582-w)
Supplement: Supplementary file 1 — Supplementary information. [file 41598_2020_66582_MOESM1_ESM.pdf]

supplementary information:

Full-length transcript sequencing accelerates the transcriptome research of *Gymnocypris namensis*, an iconic fish of the Tibetan Plateau

Hui Luo[1,2†], Haiping Liu[4†], Jie Zhang[1†], Bingjie Hu[1], Chaowei Zhou[1,2], Mengbin Xiang[1], Yuejing Yang[1,2], Mingrui Zhou[1,2], Tingsen Jing[1,2], Zhe Li[1], Xinghua Zhou[1,2], Guangjun Lv[1,2], Wenping He[1,2], Benhe Zeng[4], Shijun Xiao[3*], Qinlu Li[1*], Hua Ye[1,2*]

[1]Key Laboratory of Freshwater Fish Reproduction and Development (Ministry of Education), Southwest University College of Animal Sciences, Chongqing, 402460, China

[2] Key Laboratory of Aquatic Science of Chongqing 400175, China

[3] Department of Computer Science, Wuhan University of Technology, Wuhan, 430070, China

[4] Institute of Fisheries Science, Tibet Academy of Agricultural and Animal Husbandry Sciences, Lhasa 850000, China

S. Xiao (✉), e-mail: shijun_xiao@163.com

Q. Li (✉), e-mail: lu_8677@163.com

H. Ye (✉), e-mail: yhlh2000@126.com

[†] Hui Luo, Haiping Liu, and Jie Zhang contributed equally to this work

Table S1 The euKaryotic Ortholog Groups (KOG) classification analysis of 49138 genes.

Class	Abbreviation	Gene Numbers	Ratio
Signal transduction mechanisms	T	7696	15.66%
General function prediction only	R	7271	14.80%
Posttranslational modification, protein turnover, chaperones	O	5710	11.62%
Intracellular trafficking, secretion, and vesicular transport	U	3924	7.99%
Carbohydrate transport and metabolism	G	3739	7.61%
Cytoskeleton	Z	3400	6.92%
Transcription	K	3002	6.11%
Translation, ribosomal structure and biogenesis	J	2535	5.16%
RNA processing and modification	A	2360	4.80%
Energy production and conversion	C	2319	4.72%
Function unknown	S	1910	3.89%
Lipid transport and metabolism	I	1853	3.77%
Inorganic ion transport and metabolism	P	1449	2.95%
Amino acid transport and metabolism	E	1416	2.88%
Cell cycle control, cell division. chromosome partitioning	D	1300	2.65%
Chromatin structure and dynamics	B	778	1.58%
Nucleotide transport and metabolism	F	746	1.52%
Secondary metabolites biosynthesis, transport and catabolism	Q	740	1.51%
Defense mechanisms	V	695	1.41%
Replication, recombination and repair	L	640	1.30%
Extracellular structures	W	580	1.18%
Coenzyme transport and metabolism	H	519	1.06%
Cell wall/membrane/envelope biogenesis	M	461	0.94%
Nuclear structure	Y	182	0.37%
Cell motility	N	58	0.12%
Unamed protein	X	1	0.00%

Table S2 Gene Ontology (GO) classification analysis of genes.

## Total annotated genes: 63926			
#GO ID (Lev2)	GO Term (Lev2)	GO Term (Lev1)	Gene Number
GO:0019012	virion	Cellular Component	160
GO:0044425	membrane part	Cellular Component	18224
GO:0044456	synapse part	Cellular Component	317
GO:0031974	membrane-enclosed lumen	Cellular Component	3151
GO:0005623	cell	Cellular Component	35769
GO:0055044	symplast	Cellular Component	1
GO:0009295	nucleoid	Cellular Component	9
GO:0044422	organelle part	Cellular Component	11910
GO:0045202	synapse	Cellular Component	411
GO:0043226	organelle	Cellular Component	23955
GO:0030054	cell junction	Cellular Component	1034
GO:0044421	extracellular region part	Cellular Component	2407
GO:0032991	macromolecular complex	Cellular Component	11156
GO:0005576	extracellular region	Cellular Component	4117
GO:0044423	virion part	Cellular Component	160
GO:0044217	other organism part	Cellular Component	4
GO:0016020	membrane	Cellular Component	20620
GO:0044215	other organism	Cellular Component	4
GO:0099080	supramolecular complex	Cellular Component	1347
GO:0044464	cell part	Cellular Component	35766
GO:0044699	single-organism process	Biological Process	33122
GO:0023052	signaling	Biological Process	8876
GO:0001906	cell killing	Biological Process	13
GO:0032501	multicellular organismal process	Biological Process	12783
GO:0022414	reproductive process	Biological Process	437
GO:0050789	regulation of biological process	Biological Process	17827
GO:0040011	locomotion	Biological Process	2280
GO:0032502	developmental process	Biological Process	12841
GO:0071840	cellular component organization or biogenesis	Biological Process	11958
GO:0098743	cell aggregation	Biological Process	4
GO:0048511	rhythmic process	Biological Process	120
GO:0099531	presynaptic process involved in chemical synaptic transmission	Biological Process	132
GO:0000003	reproduction	Biological Process	441
GO:0048518	positive regulation of biological process	Biological Process	4632
GO:0065007	biological regulation	Biological Process	21584
GO:0040007	growth	Biological Process	1591

GO:0008152	metabolic process	Biological Process	29816
GO:0098754	detoxification	Biological Process	28
GO:0051179	localization	Biological Process	11406
GO:0050896	response to stimulus	Biological Process	14131
GO:0009987	cellular process	Biological Process	39021
GO:0022610	biological adhesion	Biological Process	1361
GO:0048519	negative regulation of biological process	Biological Process	4303
GO:0051704	multi-organism process	Biological Process	1502
GO:0007610	behavior	Biological Process	393
GO:0002376	immune system process	Biological Process	3525
GO:0003824	catalytic activity	Molecular Function	23696
GO:0060089	molecular transducer activity	Molecular Function	1699
GO:0045182	translation regulator activity	Molecular Function	19
GO:0005215	transporter activity	Molecular Function	4279
GO:0005488	binding	Molecular Function	33805
GO:0001071	nucleic acid binding transcription factor activity	Molecular Function	1660
GO:0016530	metallochaperone activity	Molecular Function	24
GO:0005198	structural molecule activity	Molecular Function	2154
GO:0016209	antioxidant activity	Molecular Function	205
GO:0004871	signal transducer activity	Molecular Function	1923
GO:0098772	molecular function regulator	Molecular Function	2697
GO:0000988	transcription factor activity, protein binding	Molecular Function	664

Table S4 The hit homologs ratio between NGS-based transcriptome and PacBio-based transcriptome.

Fish	pacbio seq Nunber	hit to NGS	Ratio (%)	NGS seq number	hit to Pacbio	Ratio (%)	NCBI Transcriptome reference ID
Gymnocypris namensis	125396	121,535	96.921	84,464	61,196	72.452	GHYH00000000
Gymnocypris selincuoensis	125396	121,319	96.749	106,851	73,232	68.537	GHYI00000000
Gymnocypris pzewalskii	125396	121,567	96.946	78,762	58,744	74.584	GHYJ00000000
Gymnocypris eckloni	125396	121,559	96.94	87,248	62,607	71.758	GHYG00000000

Table S5 The proportion of NGS-based transcriptome of Gymnocypris species that are annotated by homology search against Genbank NR database.

Fish	NCBI Transcriptome reference ID	Term	all sequence	NR annotated	Ratio (%)
Gymnocypris eckloni	GHYG00000000	all	87,248	34,800	39.89%
		PacBio hit*	62,607	32,167	51.38%
		PacBio not hit*	24,641	2,633	10.69%
Gymnocypris pzewalskii	GHYJ00000000	all	78,762	33,409	42.42%
		PacBio hit	58,744	31,144	53.02%
		PacBio not hit	20,018	2,265	11.31%
Gymnocypris namensis	GHYH00000000	all	84,464	34,572	40.93%
		PacBio hit	61,196	32,153	52.54%
		PacBio not hit	23,268	2,419	10.40%
Gymnocypris selincuoensis	GHYI00000000	all	106,851	37,616	35.20%
		PacBio hit	73,232	34,847	47.58%
		PacBio not hit	33,619	2,769	8.24%

* Pacbiohit and PacBio not hit represented homologs that were identified and unidentified among PacBio-based transcriptome of *G .namensis*, respectively.

Table S6 Comparison with NGS-based transcriptome and PacBio-based transcriptome of *G. namensis*

Statistics	Novel transcriptome	Previous transcriptome*
Transcript number	125,396	84,464
N50 (bp)	2,044	1,825
N90 (bp)	1,083	566
Mean (bp)	1,819	1,267
number>=2,000bp	41,248	14,864
Total length (bp)	228,095,655	107,087,800

* The references for NGS-based transcriptome used for G. namensis was from the NCBI with accession numbers of GHYH00000000.

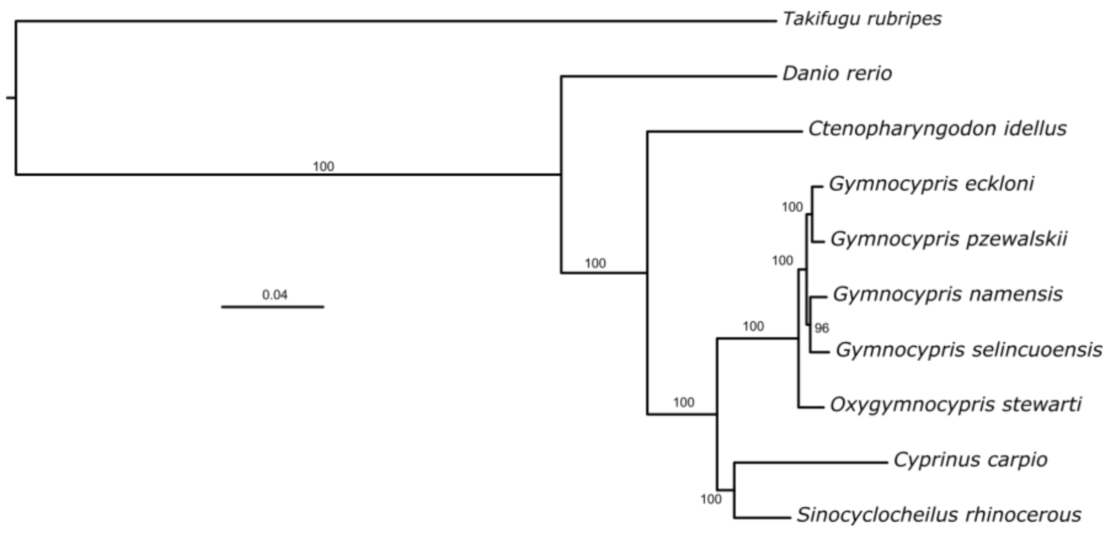

Figure S1. Phylogenetic relationships of *Gymnocypris namensis*.
